# Authorship Proliferation of Research Articles in Top 10 Orthopaedic Journals: A 70-Year Analysis

**DOI:** 10.5435/JAAOSGlobal-D-21-00098

**Published:** 2021-09-02

**Authors:** Ellen Lutnick, Antonio Cusano, David Sing, Emily J. Curry, Xinning Li

**Affiliations:** From the University at Buffalo, Jacobs School of Medicine and Biomedical Sciences, Buffalo, NY (Ms. Lutnick); the Department of Orthopedic Surgery, University of Connecticut, Farmington, CT (Dr. Cusano); and the Department of Orthopaedic Surgery, Boston University, Boston, MA (Dr. Sing, Ms. Curry, Dr. Li).

## Abstract

Scholarly impact has been used to measure faculty productivity and academic contribution throughout academia. Traditionally, the number of articles authored has been the primary metric for scholarly impact regarding academic promotion and reputation. We hypothesize that over time, the nature of authorship has evolved to include more authors per research article throughout the history of orthopaedic literature.

**Methods:**

Bibliometric data for all original research article abstracts were extracted from PubMED for the 10 highest rated H5-index orthopaedic clinical journals (“*American Journal of Sports Medicine*,” “*Journal of Bone and Joint Surgery American Volume*,” “*Clinical Orthopaedics and Related Research* “*Spine*,” “*Knee Surgery, Sports Traumatology, Arthroscopy*,” “*Journal of Arthroplasty*,” “*Arthroscopy*,” “*The Spine Journal*,” “*European Spine Journal*,” and “*Journal of Bone and Joint Surgery British Volume/Bone & Joint Journal*”). The number of authors per article was then analyzed over time using the Cochran-Armitage trend test.

**Results:**

A total of 106,529 original articles were analyzed over a 70-year period. The number of authors increased significantly over time from a mean of 1.4 authors (SD: 0.62) in 1946 to 5.7 authors (SD: 3.1) in 2019, representing an average relative increase of 4.3% per year (*P* < 0.05). The three oldest journals had the lowest average authors (*Journal of Bone and Joint Surgery Am Volume*: 1946, mean 3.7 authors [SD: eight]; *Journal of Bone and Joint Surgery Br Volume/Bone & Joint Journal*: 1948, mean: 3.6 authors [SD: 7.5]; *Clinical Orthopaedics and Related Research*: 1963, mean 3.3 authors [SD: 2.9]). The three newest journals had the highest average authors (*European Spine Journal*: 1992, mean 5.3 authors [SD: 3.3]; *Knee Surgery, Sports Traumatology, Arthroscopy*: 1993, mean 5.5 authors [SD: 6.7 authors; *The Spine Journal*: 2003, mean 5.2 authors [SD: 3.6]).

**Discussion:**

Original research articles published in orthopaedic academic journals have experienced an increase in authorship over time. Although our data cannot explain what has driven this change, increasing cooperation between collaborators may represent less contribution per author over time.

Scholarly impact has been used to measure faculty productivity and reputation within academia. Traditionally, the number of authored articles has served as the primary metric for determining academic promotion, in addition to involvement in clinical care and education. Over the past decade, however, there has been increased consideration for other areas of development, such as innovation, quality improvement projects, informatics, and digital scholarship including social media.^1^ Although these new disciplines have redefined the scope of scholarly work, research productivity as measured by the number of publications is still universally used to quantitively evaluate one's academic successes.

A recent analysis of approximately 24 million PubMed-indexed papers published between 1913 and 2013 showed an exponential trend in the number of published works over the last century, with more than a five-fold increase in the average number of authors per manuscript over this timeframe.^2^ This trend has been well established among medical journals^3,4^ and is likely due, in part, to both the increasing complexity of modern research, including multicentered trials and the need for productivity for grounds of academic promotion.^5^ However, our understanding of authorship because it applies to orthopaedic surgery literature is limited.

Academic productivity can be quantified in a variety of ways. Classically, one's total number of publications and citations were used, although both can be disproportionately affected by one's participation in a single publication of major influence in a high-quality journal or in the production of poor-quality work in a low-quality journal, respectively. To address the main pitfalls of using these bibliometric indicators alone, the H-index was created, which considers an author's list of published works ranked in a descending order by the number of times they are individually cited. The maximum value of one's H-index is equal to the number of papers (N) that each has at least that same number (N) of citations. Under this calculation, if an author's H-index is 10, then it means 10 of their publications each have at least 10 citations and all remaining works have less than 10 citations. Similarly, if a more productive researcher has an H-index of 40, it means that he or she has 40 publications that each has at least 40 citations. In this way, the H-index attempts to quantify both the academic productivity of an author and the effect of their work in a single metric. Although the H-index does not directly quantify the quality of an author's publications, it relies on the premise that an author's publications that have been cited more times are likely to be of higher quality or related to a topic that is possibly more original or of greater import. Authorship patterns have recently been scrutinized throughout medical subspecialties to identify the prevalence of ghost authorship, better understand the effect of particular demographic data on research outcomes, and to evaluate the implications of medical student involvement on a principal investigator's H-index or educational promotions over time.^6,7,8,9,10^

Although the number of co-authors has increased over time in other medical and surgical specialties, this trend has not been comprehensively evaluated because it applies to the field of orthopaedic surgery. Thus, this study sought to (1) characterize bibliographic trends in orthopaedic surgery among highly productive orthopaedic researchers and (2) determine how scholarly impact measures including publication count, citation count, and H-index apply to academic orthopaedic literature.

## Methods

### Data Source

Bibliometric data for 106,529 original research article abstracts (not including systematic reviews, narrative reviews, and case reports) published from 1946 to 2019 were extracted from PubMed from the 10 highest H5-indexed (H-index for articles published over the last 5 complete years) orthopaedic clinical journals: *American Journal of Sports Medicine* (AJSM); *Journal of Bone and Joint Surgery Am Volume*; *Clinical Orthopaedics and Related Research*; *Spine*; *Knee Surgery, Sports Traumatology, Arthroscopy*; *Journal of Arthroplasty* (JOA); *Arthroscopy*; *The Spine Journal*; *European Spine Journal*; and the *Journal of Bone and Joint Surgery British Volume/Bone & Joint Journal*, as highlighted in Table 1. Extracted data elements included PubMed ID, journal name, article title, type of article, date of publication, and the complete author listing. From this extracted data, the number of authors credited per article was calculated and stratified by publication year and the published journal.

**Table 1:** Average Number of Authors per Article in the top 10 Orthopaedics Journals^a^

Rank by Productivity	Journal	Year First Published	Average No. of Authors		Productivity/Citation Impact	
			Median (IQR)	Mean (SD)	H5-Index	H5-Median
1	*The American Journal of Sports Medicine*	1976	5 (3.6)	4.9 (4.4)	88	112
2	*The Journal of Bone and Joint Surgery, American Volume*	1946	3 (2.5)	3.7 (8)	78	102
3	*Clinical Orthopaedics and Related Research*	1963	3 (2.5)	3.3 (2.9)	72	91
4	*Spine*	1978	5 (3.6)	5 (3.1)	66	88
5	*Knee Surgery, Sports Traumatology, Arthroscopy*	1993	5 (4.7)	5.5 (6.7)	63	78
6	*The Journal of Arthroplasty*	1986	5 (3.6)	4.7 (2.6)	62	81
7	*Arthroscopy*	1985	4 (3.6)	4.4 (2.3)	61	91
8	*The Spine Journal*	2003	5 (3.6)	5.2 (3.6)	55	79
9	*European Spine Journal*	1992	5 (3.6)	5.3 (3.3)	55	69
10	*The Journal of Bone and Joint Surgery, British Volume/Bone & Joint Journal*	1948	3 (2.5)	3.6 (7.5)	54	70

All statistics current as of June 2019.

### Authorship Analysis

**Figure 1:**
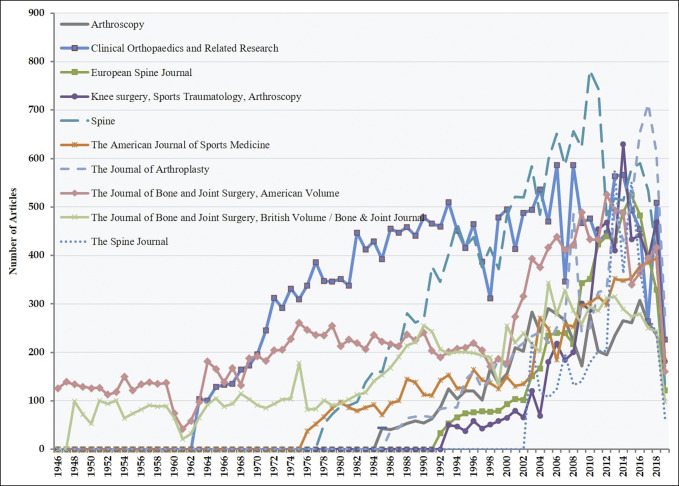
Graph showing number of articles per year in each of the top 10 orthopaedic journals

**Figure 2:**
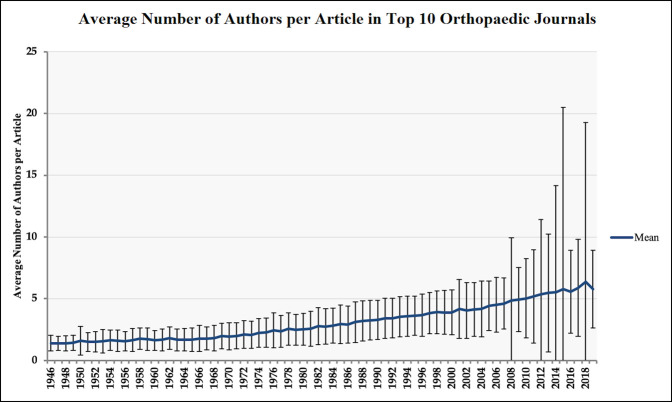
Graph showing average number of authors per article in the top ten orthopaedic journals

The number of articles published and their respective author list for each of the aforementioned top 10 H5-indexed orthopaedic journals was analyzed from 1946 to 2018. Calculations were made to determine the average number of authors by journal for each year to highlight trends over this period. Figure 1 illustrates the number of articles published in each of these top 10 journals over this period while Figure 2 illustrates the average number of authors per article over the same time.

### “Highly Productive” Author Analysis

**Figure 3:**
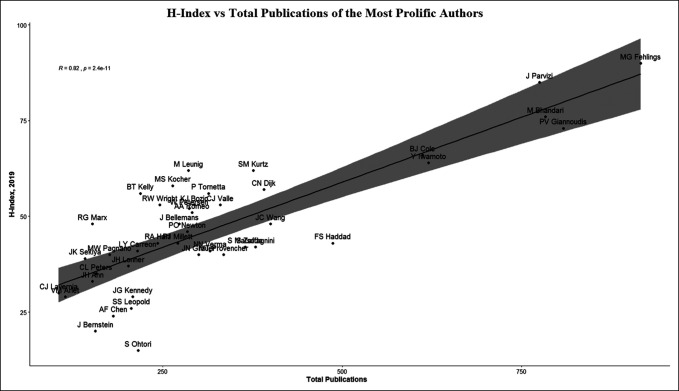
Graph showing H-index versus total publications of the most prolific authors

**Figure 4:**
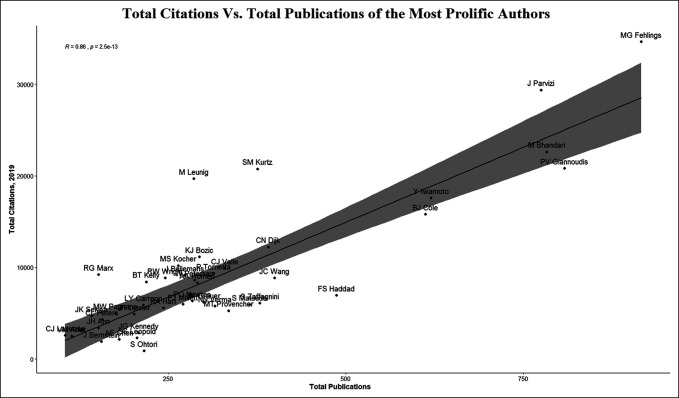
Graph showing total citations versus total publications of the most prolific authors

A comprehensive list of first and last authorships for each author was created to identify the top 250 most productive authors over this period. Authors were included if they first published research starting between 1995 and 1999. The mean and median number of authors credited per article published, their respective H-index, total publication count, and total citation count as of 2019 per Scopus were collected. These variables were compared to determine the reliability of these commonly used metrics because they apply to academic productivity. Figure 3 shows the comparison of the H-index and the total publications of these authors, and Figure 4 shows the comparison of the total citations and the total publications of this cohort.

### Statistical Testing

The number of authors per article was analyzed over time using the Cochran-Armitage trend test with a *P* value < 0.05 considered statistically significant. All statistical analyses were done using R 3.0.2 (R Foundation, www.r-project.org).

## Results

A total of 106,529 original articles were included in analysis. When stratified by publication year, a significant increase in published articles was appreciated within the aforementioned orthopaedic journals over the last 70 years (Figure 1). The number of authors per article increased significantly over this period, from a mean of 1.4 authors (SD: 0.62) in 1946 to 5.7 authors (SD: 3.1) in 2019, representing an average relative increase of 4.3% per year (*P* < 0.05) (Figure 2). The three most long-standing journals had the lowest average number of included authors over this period (*Journal of Bone and Joint Surgery Am Volume*: 1946, mean 3.7 authors [SD: 8]; *Journal of Bone and Joint Surgery Br Volume/Bone & Joint Journal*: 1948, mean: 3.6 authors [SD: 7.5]; *Clinical Orthopaedics and Related Research*: 1963, mean 3.3 authors [SD: 2.9]), whereas the three newest journals had the highest average number of authors (*European Spine Journal*: 1992, mean 5.3 authors [SD: 3.3]; *Sports Traumatology, Arthroscopy*: 1993, mean 5.5 authors [SD: 6.7 authors; *The Spine Journal*: 2003, mean 5.2 authors [SD: 3.6]).

Of the 30 articles with the highest number of authors credited for contribution (average: 184 authors per publication, range: 69 to 911), 26 articles included large multicenter trials, such as the Fracture Fixation in the Operative Management of Hip Fractures, Multicenter Orthopaedic Outcomes Network, the Study to prospectively evaluate reamed intramedullary nails in patients with tibial fractures, the MultiCenter anterior cruciate ligament (ACL) Revision Study study groups (average of 192 authors per publication), and four articles from the Science of Variation Group (average of 134 authors per publication). All 30 of these articles were published within the last 7 years.

Of the 250 “highly prolific” orthopaedic authors identified in the aforementioned journals, there were 42 who began publishing between 1995 and 1999, 22/42 of which had H-indices that fell below the mean (52%), and 20/42 had H-indices that fell above the mean (48%) (Figure 3). In a separate analysis, outliers, defined as those with H-indices that fell more than 1 SD above or below the mean were identified and examined. This analysis found 15 outliers (36%) with H-indices more than 1 SD above the mean and 12 outliers (29%) 1 SD below the mean. For total publication count, we found that 21/42 (50%) of these authors had total citations that fell below the mean while 21 (50%) had total citations that fell above the mean (Figure 4).

## Discussion

Our main findings show an increased number of authors credited per published article among the top 10 H5-indexed orthopaedic journals from 1946 to 2019. This trend is consistent with what has been previously described in other fields of medicine. Namely, a study of authorship patterns in prestigious US medical journals from 1980 to 2000 including the Annals of Internal Medicine, Archives of Internal Medicine, Journal of the American Medical Association, and The New England Journal of Medicine found an average increase in authors per article from 4.5 in 1980 to 6.9 in 2000.^3^ Similarly, our data in the orthopaedic literature also report a relative increase from a mean of 1.4 authors per publication in 1946 to 5.7 in 2019, altogether representing an increase of 4.3% per year. The rate of group authorship increased from virtually 0% to over 15% during this timeframe, with group authorship being most prevalent in journals that placed restrictions on the total number of authors allowed per manuscript. The proportion of manuscripts with a single-listed author during this timeframe decreased from approximately 4% to 1%.^3^ Although the increase in authorship may be influenced by a recent focus on large multicenter trials to better guide clinical practice, our data call into question the current approaches used to objectively quantify one's scholarly impact based on the number of publications within academic medicine.

The increase in authorship in orthopaedic surgery is partly secondary to the emergence of large-scale clinical multicentered trials and group authorship. All 30 articles with the greatest number of cited authors were part of larger collaborative research efforts, and 26/30 were large multicenter trials, including those conducted by the Fracture Fixation in the Operative Management of Hip Fractures, Multicenter Orthopaedic Outcomes Network, Study to prospectively evaluate reamed intramedullary nails in patients with tibial fractures, fluid lavage of open wounds (FLOW) and MultiCenter ACL Revision Study research groups (Appendix 1, http://links.lww.com/JG9/A151). Defining authorship in these larger, collaborative works can be hard to quantify, causing many to challenge whether various nontraditional roles such as patient recruitment should continue to be considered legitimate grounds for authorship. Authorship-by-committee models have been proposed to allow for individuals of a larger collaborative effort to be collectively credited. To that end, four of the aforementioned 30 works were a product of the Science of Variation Group, which involves polling specialists about how they interpret data or diagnose problems.^11,12,13,14^ In this case, each polled specialist was credited with authorship for their contribution to the final paper. However, this survey type of group research is distinctly different from research groups conducting multicenter trials, wherein authors have more extensive and traditional responsibilities to the data collection, interpretation, or presentation, for example. Some of the increasing collaboration demonstrated by our analysis may possibly be related to the changing nature of clinical orthopaedic practice from private practice consolidation into more academic settings, which allows for easier access to the number of potential contributors and the ability to obtain grant funding, tending to favor applications with a history of group collaboration.

Another possible explanation for increases in authorship includes more medical student involvement in research. Wickramasinghe et al^9^ retrospectively reviewed PubMed and Scopus with the keyword “medical student” and found an exponential increase in medical student research from 1980 to 2010, with medical students listed as the first author in 170 studies (48.6%) and 55 studies authored by a single medical student. The three most common areas of medical student involvement in research, in descending order, were psychiatry, general medicine, and medical education, with most of these articles (n = 207, 59.1%) never being cited. Although the numbers of medical student authors per publication have remained static, the total numbers of authors have consequently increased because of this.^9^ Medical student involvement within orthopaedics is likely a sequela of orthopaedic residencies being extremely competitive to match into, drawing medical students toward research as a way to bolster their application and resume. Sachs et al retrospectively reviewed student authorship rates from 2006 to 2014 in the Journal of the American College of Surgeons to determine whether the H-index of corresponding authors changed based on the involvement or absence of student authors. Although the student authorship and first or second student author rates doubled over this period, it was without detriment to the corresponding authors' H-index or scholarly advancement. As such, the authors concluded that involving students in surgical research should be encouraged.^10^

To better assess authorship qualification for manuscripts, several medical journals require submission of authorship involvement to ensure that certain criteria for authorship have been met. Most of the journals included in our article use the International Committee of Medical Journal Editors (ICMJE) Recommendations, which attempt to standardize the ethics, preparation, and formatting of manuscripts submitted to biomedical journals for publication. Their requirements specifically call for authorship based on criteria including making “substantial contributions to the conception or design of the work; the acquisition, analysis, or interpretation of data for the work; drafting the work or revising it critically for important intellectual content; or offering final approval or the version to be published,” for example. The ICMJE further specifies that the sole acquisition of funding or collection of data and general supervision does not justify authorship and that all contributing authors are subject to accountability for all aspects of the work in the instance that the integrity or accuracy of any part of the work be investigated. In 2014, the Committee on Publication Ethics released an official document discussing what constitutes authorship and concluded that all journals should have a basic policy clearly stated in the journal's “Information for Authors” page on what appropriately constitutes authorship versus an acknowledgment instead. They further recommend that a statement of each individual's contribution to the publication be collected and included that authors assume responsibility for the integrity of the manuscript itself. With the exception of the *Journal of Arthroplasty* and *Spine Journal*, which has their own authorship guidelines, all other included journals in this study adhere to the ICJME recommendations. In addition, JOA and *Spine J* have additional specifications regarding ghost authorship, group authorship, order of authors on the publication, and contributor status through acknowledgments. AJSM has its own original Author Disclosure Statement, although it alternatively accepts the ICMJE disclosure form along with the AJSM supplemental form.

Although these criteria for authorship are meant to ensure that credit is appropriately assigned, they have certainly not eliminated all controversy surrounding the authorship criteria. First, the criterion of requiring that authors give “final approval of the version to be published” allows for the possibility of denying a deserving author access to the final version of a manuscript, or alternatively, the situation wherein an individual could withhold approval and delay/prevent the submission of a particular manuscript indefinitely. Specific journals have tried to circumvent this issue by defining an acceptable number of authors allowed per manuscript. For example, of the cohort of journals included in our study, JOA limits authorship to six authors, *Arthroscopy* to seven authors, *JBJS-Br* to eight authors, and *JBJS-Am* to six authors. The remaining journals included in our analysis do not specify a limit on the number of authors, which could potentially introduce biases and underreporting of authorship.

We identified the 42 most frequently cited authors in the included top 10 orthopaedic journals who began publishing between 1995 and 1999 and compared their relative H-indices and citation counts as of 2019. Further analysis showed that 15/42 authors (36%) had H-indices 1 SD above the mean and 12/42 authors (29%) below the mean. This information suggests that those authors whose H-indices fell above are considered to be more productive or have more academic contributions if their publication count alone were considered. Conversely, under this model, those falling 1 SD below the mean may be overlooked when solely basing productivity on the H-index alone. The H-index attempts to quantify the quality of an author's publications. For example, a lower H-index may indicate an author with many publications that are not frequently cited while a higher H-index indicates an author who published fewer papers that were more commonly cited. Our analysis reinforces the concept that the H-index as a standalone assessment tool is not a fully reflective picture of one's academic productivity. Other limitation to using H-index alone is its temporal nature, in that younger academicians who have not yet amassed a body of work with many citations are overlooked, despite their notable contributions.^15^ In an attempt to address some of its other limitations, the H-index has since been modified to better reflect an individual's academic output by incorporating authorship position in its calculation.^16^ The presented data suggest that among highly productive authors of orthopaedic literature, using H-index or citation count alone may not truly represent an author's academic productivity. Rather, a comprehensive approach to evaluating academic productivity should be considered through the use of multiple metrics including the H-index and citation count, in addition to the number of articles published per author.

Limitations of this study include the scope of our search, in that it only includes the top 10 most prolific orthopaedic journals. The patterns of these findings may change with the inclusion of more journals, including lower impact journals. In addition, because authorship guidelines are defined by individual journals, there was no way to isolate this effect from other factors that could have influenced our measures of authorship proliferation; it is possible that had these guidelines not been developed, there may have been an even larger growth in the number of authors per article over time. We were not able to categorize any specific qualities related to authorship that have changed over time or the potential influence from demographic data, such as sex, inclusion of medical student authors, or any delineation of the characteristics of authors based on their current clinical or academic standing.

Future research analyzing student authorship within the field of orthopaedics including their authorship patterns and subsequent effect on the H-index of the principal investigator is warranted, as well as analysis of the pattern of accepted orthopaedic residency applicant average publications over time to demonstrate the value of higher authorship in this demographic. In addition, analysis of the effects of surgeon compensation, the proliferation of new technologic advances, and the changing nature of clinical practice because they relate to the encouragement of increased collaboration is warranted to better understand the driving forces behind the changing nature of authorship. Finally, future efforts should also include an analysis on the role of demographics such as race and sex on academic productivity. Other fields have demonstrated a plateau in publications by women in recent years while some fields illustrate a decline in female authors, suggesting that the underrepresentation of women in high impact medical journals remains an issue.^8^ In the field of orthopaedics and sports medicine, in particular, female investigators are authoring publications at a growing rate, having increased almost sevenfold from 1972 to 2018.^6^

## Conclusion

Despite the dynamic landscape of authorship, it still remains the currency by which academicians are evaluated, and therefore, remains essential for tenure, promotion, salary, and grant funding. Our study clearly illustrates the notable increase in authorship specific to the field of orthopaedics and also suggests that the current standard of using an author's total publications regarding academic promotion may be more biased than previously thought. Authorship proliferation over time serves to highlight the need to develop other metrics to evaluate an author's comprehensive productivity, rather based purely on the number of publication count alone. The consideration of other metrics such as citation count and H-index allows for a more comprehensive, holistic assessment of an author's contribution to academia.
